# Satellites capture socioeconomic disruptions during the 2022 full-scale war in Ukraine

**DOI:** 10.1038/s41598-023-42118-w

**Published:** 2023-09-22

**Authors:** Iolanda Ialongo, Rostyslav Bun, Janne Hakkarainen, Henrik Virta, Tomohiro Oda

**Affiliations:** 1 Space and Earth Observation Centre, Finnish Meteorological Institute, Helsinki, Finland; 2 Department of Applied Mathematics, Lviv Polytechnic National University, Lviv, Ukraine; 3 Department of Transport and Computer Science, WSB University, Dąbrowa Górnicza, Poland; 4 Earth From Space Institute, Universities Space Research Association, Washington, D.C USA; 5 Department of Atmospheric and Oceanic Science, University of Maryland, College Park, MD USA; 6 Graduate School of Engineering, Osaka University, Suita-City, Osaka Japan

**Keywords:** Atmospheric science, Climate change

## Abstract

Since February 2022, the full-scale war in Ukraine has been strongly affecting society and economy in Ukraine and beyond. Satellite observations are crucial tools to objectively monitor and assess the impacts of the war. We combine satellite-based tropospheric nitrogen dioxide (NO_2_) and carbon dioxide (CO_2_) observations to detect and characterize changes in human activities, as both are linked to fossil fuel combustion processes. We show significantly reduced NO_2_ levels over the major Ukrainian cities, power plants and industrial areas: the NO_2_ concentrations in the second quarter of 2022 were 15–46% lower than the same quarter during the reference period 2018–2021, which is well below the typical year-to-year variability (5–15%). In the Ukrainian capital Kyiv, the NO_2_ tropospheric column monthly average in April 2022 was almost 60% smaller than 2019 and 2021, and about 40% smaller than 2020 (the period mostly affected by the COVID-19 restrictions). Such a decrease is consistent with the essential reduction in population and corresponding emissions from the transport and commercial/residential sectors over the major Ukrainian cities. The NO_2_ reductions observed in the industrial regions of eastern Ukraine reflect the decline in the Ukrainian industrial production during the war (40–50% lower than in 2021), especially from the metallurgic and chemical industry, which also led to a decrease in power demand and corresponding electricity production by thermal power plants (which was 35% lower in 2022 compared to 2021). Satellite observations of land properties and thermal anomalies indicate an anomalous distribution of fire detections along the front line, which are attributable to shelling or other intentional fires, rather than the typical homogeneously distributed fires related to crop harvesting. The results provide timely insights into the impacts of the ongoing war on the Ukrainian society and illustrate how the synergic use of satellite observations from multiple platforms can be useful in monitoring significant societal changes. Satellite-based observations can mitigate the lack of monitoring capability during war and conflicts and enable the fast assessment of sudden changes in air pollutants and other relevant parameters.

## Introduction

Since the beginning of the full-scale war in Ukraine in February 2022, the life of the Ukrainian population and the country’s economy have been strongly affected by the war. By June 2022 Russia occupied 20% of Ukraine's territory^1^. Almost 19,000 civilian casualties have been registered in the country after 1 year of war^2^. The United Nations High Commissioner for Refugees (UNHCR) reported that about 8 million Ukrainians (about 24% of the total population) left the country as refugees across Europe^3^. The World Bank estimates the reconstruction and recovery cost as $349 billion, which is more than 150% of Ukraine’s GDP in 2021^4^. War-induced environmental damages and risks to energy and industrial infrastructure, urban areas, agricultural and natural resources have also been discussed^5–7^. Reductions in crop harvesting have been reported near the front line^8^, which impacts global food security, as Ukraine is a major world exporter of wheat^9^. Despite the ongoing efforts by the Ukrainian authorities^10^ and other organizations^11,12^, the monitoring capabilities have been significantly limited due to lack of data and incomplete information, especially from the occupied areas, and the uncertainties associated with the impact assessments remain large. Also, the emissions from military actions are difficult to assess and are not included in traditional emission inventories^13^.

Satellite-based Earth observations can collect near real-time objective information globally including areas where ground-based data collection is difficult. Satellite observations of air pollutants such as nitrogen dioxide (NO_2_) have a history of success in monitoring the air quality levels and their improvements globally^14^. Nitrogen oxides (NO_x_ = NO + NO_2_) are mostly emitted from fossil fuel combustion processes, e.g., from coal-burning power plants, metallurgic and coke production processes, transportation, and fires. Satellite NO_2_ observations have been used to derive NO_x_ emissions and their trends, and to evaluate the success of environmental policy measures and the impacts of the global economic crises^15–19^. Such observations have also been applied to rapidly assess NO_2_ pollution levels over several cities in response to the reduced mobility and industrial activities during the COVID-19 pandemic^20–23^. Reductions in NO_2_ and ammonia have been previously reported as a consequence of the Syrian Civil War in 2011^24,25^. Similarly, over the past decade, satellite observations of greenhouse gasses (GHGs), such as CO_2_ and CH_4_, have become available and started playing a crucial role in climate mitigation monitoring (^26^ and reference therein). Recent studies have demonstrated that the combined use of satellite air quality and GHG data improves the ability of estimating surface emissions and characterizing the sources^27–29^. Combining a wide variety of satellite observations, including atmospheric concentrations and imagery data can further enhance the ability to monitor changes and attribute them to specific human and/or natural processes.

In this work, we exploit the synergy of different Earth satellite observations from multiple platforms to study the impacts of the war in Ukraine. We use the Copernicus Sentinel 5-Precursor/TROPOspheric Monitoring Instrument (S5P/TROPOMI) NO_2_ tropospheric column data^30^ to assess the variations in fossil fuel usage related to changes in population, power generation and other industrial activities in Ukraine. We also explore satellite imageries as well as fire/thermal anomaly data to characterize the signatures and the spatio-temporal distribution of combustions (e.g., shelling and other fires) near the front line. We also consider column-averaged CO_2_ dry air mole fraction (XCO_2_) data, collected from NASA’s OCO-2 (Orbiting Carbon Observatory-2) satellite to analyze persistent CO_2_ anomalies.

## Results

### Substantial NO_2_ reductions observed over Ukraine

**Figure 1:**
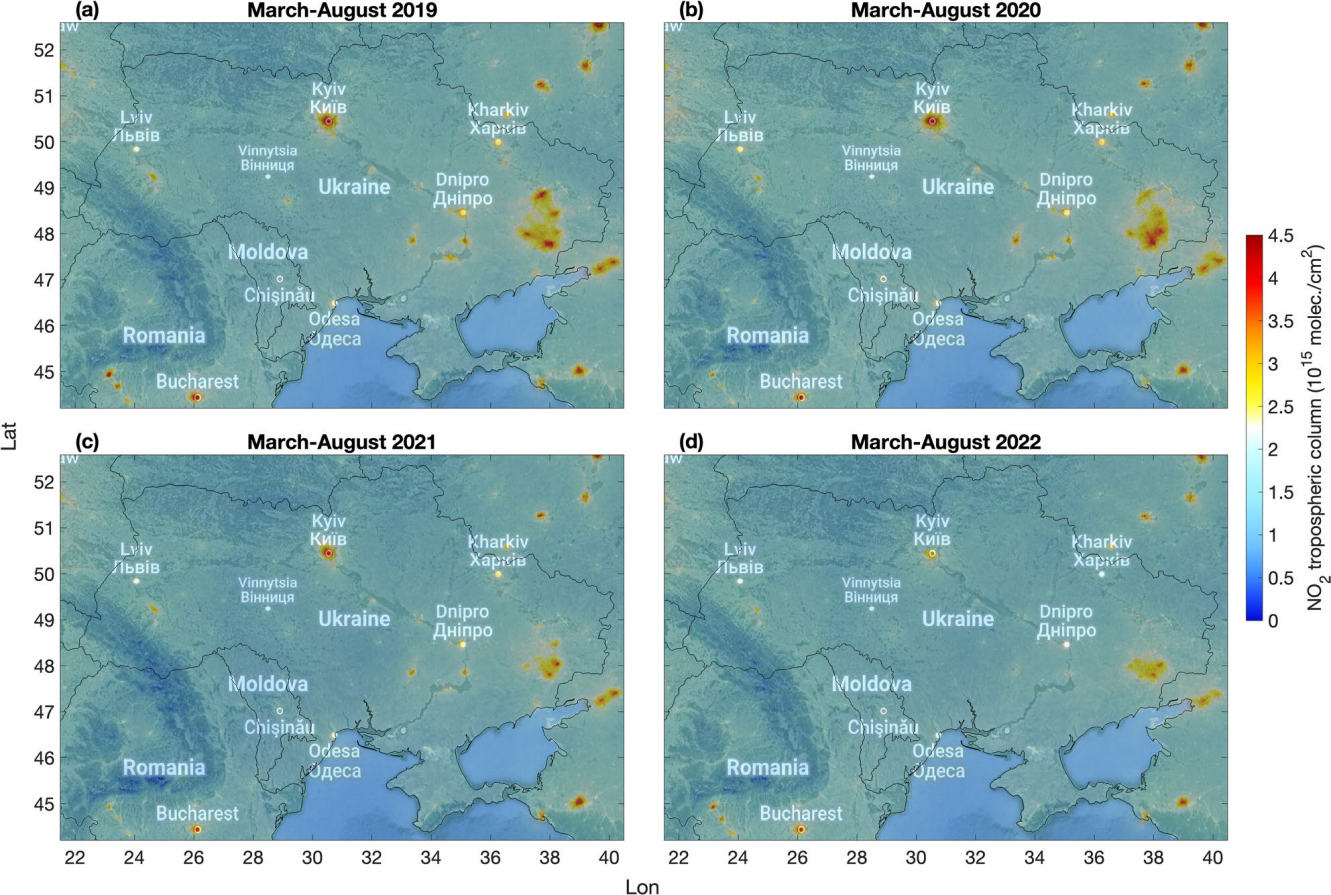
Average S5P/TROPOMI NO_2_ maps over Ukraine. March–August average tropospheric NO_2_ columns for each year between 2019 and 2022. The maps were generated using the Matlab tool plot_google_map (Version 2.0.0.1, https://github.com/zoharby/plot_google_map) with administrative boundary shapefile from https://www.naturalearthdata.com/downloads/10m-cultural-vectors/10m-admin-0-countries/ (Version 3.1.0).

We observed substantial NO_2_ reductions over many major urban and industrial areas in Ukraine (Supplementary Fig. 1) from March to August 2022 as compared to previous years (Fig. 1). The observed reductions of NO_2_ are consistent in timing and location with the reported reductions in traffic, commercial and residential activities in the cities as well as from slowed down power and heat production and industrial activities during the war^31^. Correspondingly, muted nighttime lights (NTL) have been observed over several Ukrainian cities^32,33^ by the VIIRS satellite nighttime imagery from the Day/Night Band (DNB, 500–900 nm). NO_2_ reductions as compared to previous years are observed for example over the capital city of Kyiv (Supplementary Fig. 2) and in the area around the city of Donetsk in south-eastern Ukraine (Supplementary Fig. 3). Kyiv was attacked during the first phase of the war (February–March 2022) and a significant part of the population left the city at that time but partially returned by July 2022^34^. Near the city of Donetsk, the largest NO_2_ enhancements correspond to five coal-fired power plants (Supplementary Fig. 3). This area was partially occupied by Russia in 2014 (Supplementary Fig. 1). Two power plants are controlled by Russia (Starobeshivska and Zuyivska, with capacity of 2010 MW and 1220 MW, respectively) and two by Ukraine (Kurakhivska and Slovyanska, with capacity of 1532 MW and 830 MW, respectively). In addition, the Vuhlehirska power plant (capacity 3600 MW) was occupied by Russia on 26 July 2022^35^. The NO_2_ levels observed in 2022 over the power plants controlled by Russia since 2014 remain closer to those of the previous years, while the other facilities experienced more substantial reductions. These large coal-fired power plants are located a short distance from the front line and might have experienced disruptions already since 2014, but possible changes in NO_2_ before 2022 remain closer to the normal year-to-year variability. In 2021, there was also a period with critically low coal reserves at the thermal power plants controlled by Ukraine, which did cause a decrease in electricity generation for example from the Vuhlehirska power plant^36^. There are several large metallurgical and coke plants near Donetsk (see Supplementary Fig. 1) but they had a lower impact on the overall NO_2_ patterns (Supplementary Fig. 3).

**Figure 2:**
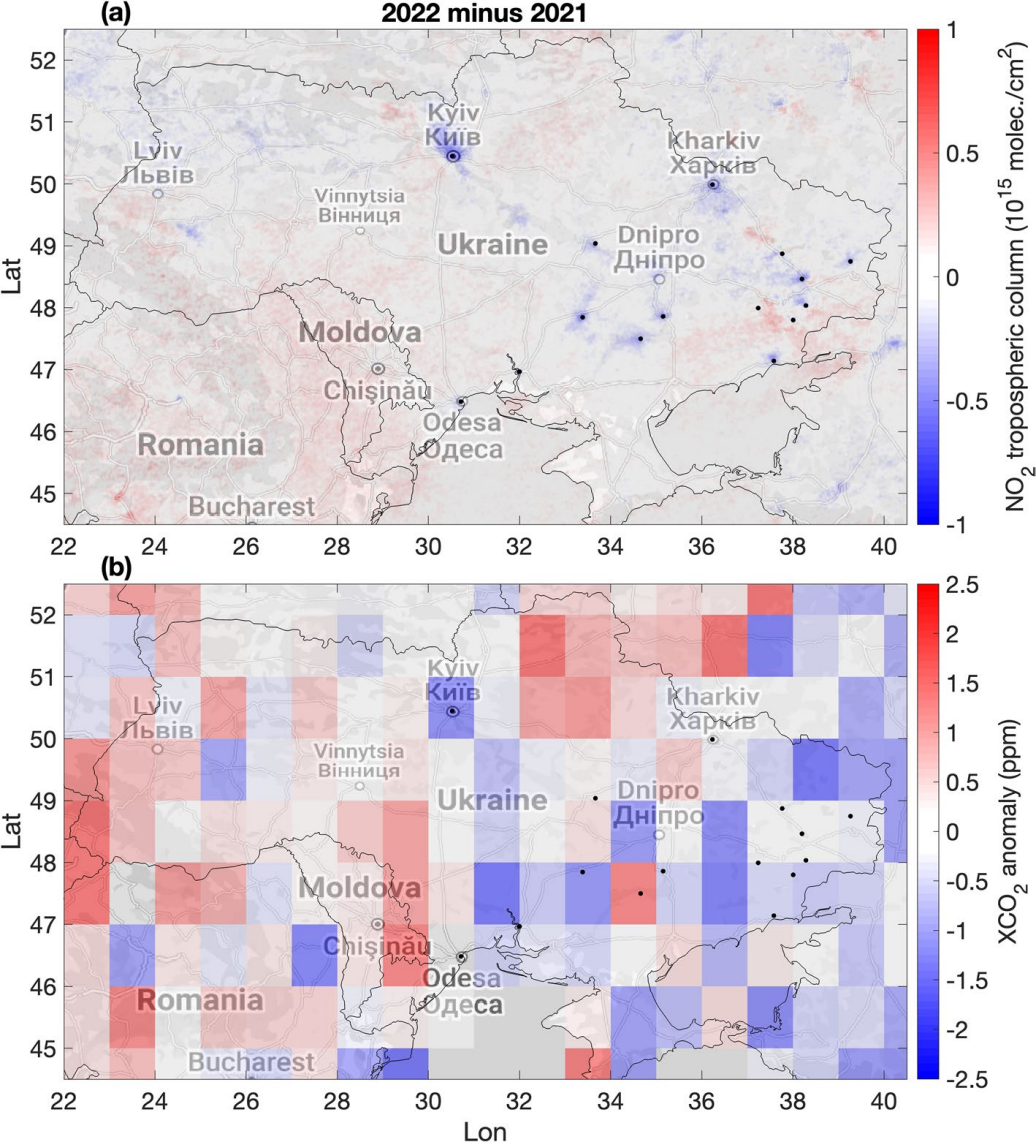
Differences in NO_2_ and XCO_2_ anomalies between 2022 and 2021 over Ukraine. (**a**) Difference of the March–August mean tropospheric NO_2_ columns between 2022 and 2021 based on S5P/TROPOMI observations. (**b**) Difference of the March–August mean XCO_2_ anomalies (defined as the difference from the latitudinal background) between 2022 and 2021 based on OCO-2 observations. Blue colors indicate reductions observed in 2022. Black dots correspond to the major cities, industrial areas and power plants. The maps were generated using the Matlab tool plot_google_map (Version 2.0.0.1, https://github.com/zoharby/plot_google_map) with administrative boundary shapefile from https://www.naturalearthdata.com/downloads/10m-cultural-vectors/10m-admin-0-countries/ (Version 3.1.0).

The NO_2_ levels were also strongly reduced over the Dnipro-Zaporizhzhia-Kryvyi Rih region (Supplementary Fig. 4), including the contribution from Zaporizhzhia’s industrial area (in particular Zaporizhkoks coke and ArcelorMittal steel plant); Enerhodar, where the coal-burning Zaporizka thermal power plant is located; Kryvyi Rih, including coke and the Inhuletskyi ore beneficiation plant in the south and the Ternivskyi District in the north (the largest in Europe Pivnichnyi ore beneficiation plant, and Thentralnyi ore beneficiation plant); Dnipro city (steel and cast iron production); Kamianske, hosting both heavy and chemical industry; Horishni Plavni, a purpose-built iron mining city. The NO_2_ difference map (Fig. 2a) further highlights the localized reductions in NO_2_ levels in 2022 as compared to 2021 over the impacted areas.

**Figure 3:**
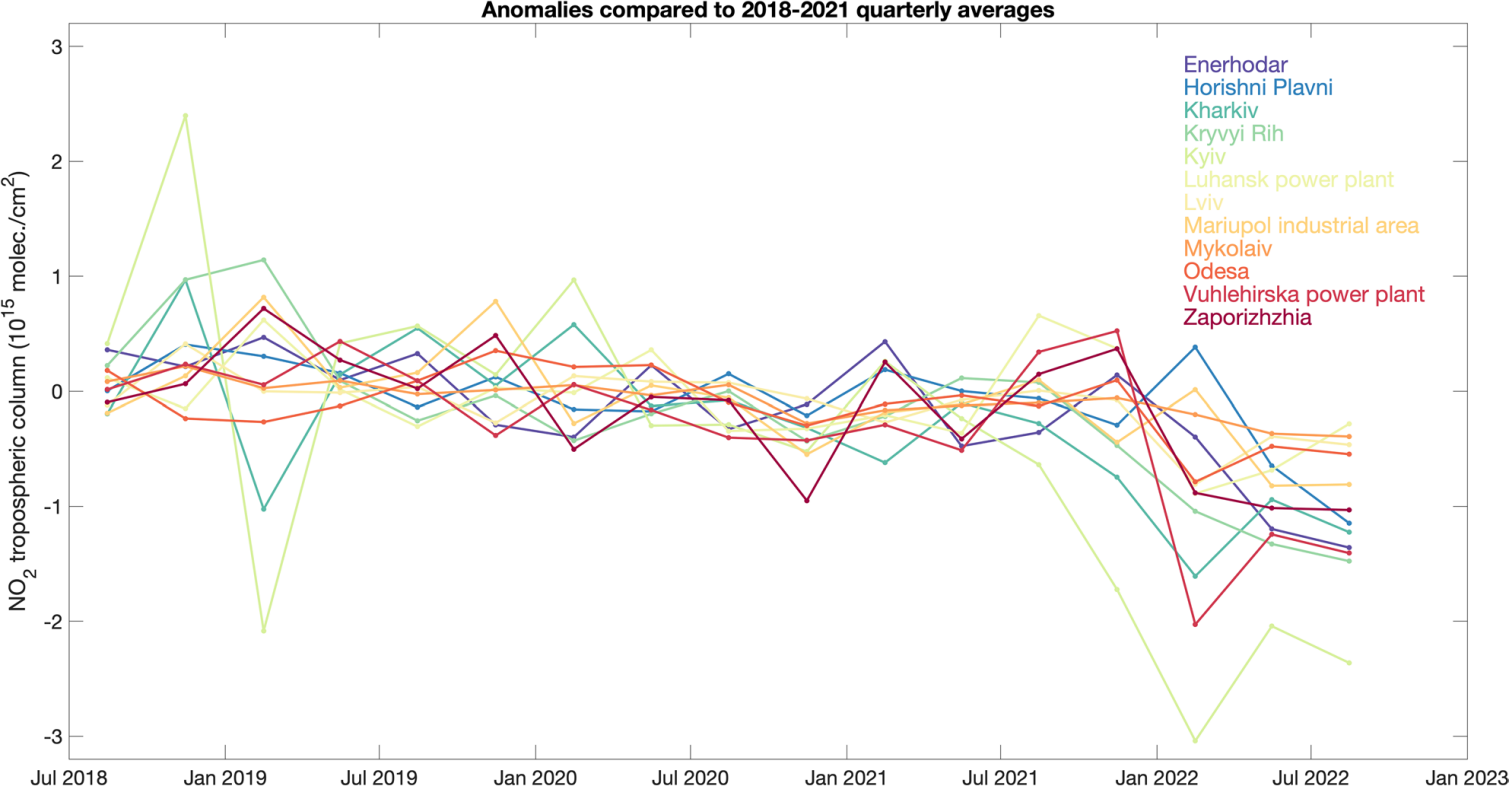
Timeseries of quarterly NO_2_ anomalies for several cities and industrial facilities in Ukraine. The anomalies are defined as the difference of the quarterly mean of individual years and the quarterly mean for the reference period 2018–2021.

In 2022 the quarterly mean NO_2_ concentrations were well below the quarterly mean levels from the reference period (2018–2021) and the NO_2_ reductions were larger than the typical variability (5–15%) at most locations (Fig. 3). The NO_2_ concentrations over the major cities and emission areas in the second quarter of 2022 were 15–46% lower than the same quarter during the reference period 2018–2021 (Table 1). A slight gradual reduction in the NO_2_ levels is apparent since 2018 at some locations (Fig. 3). In addition to changes in emissions, the observed year-to-year variability can partly depend on meteorological factors, especially related to changes in wind conditions^21^. When considering only calm wind conditions (e.g., wind speed smaller than 3 m/s), which reduces the effect of NO_2_ outflow away from the source, we find that the NO_2_ reductions in the second quarter of 2022 become even larger (Supplementary Fig. 5, Table 1) as compared to the reference period. One exception is the city of Lviv, for which a 3% increase in NO_2_ concentrations is observed in the second quarter of 2022 under calm wind conditions (instead of 15% reduction under all wind conditions). Lviv, located near the western border with Poland, did not experience reductions in population as other Ukrainian cities and was hosting 240,000 internally displaced persons as of July 2022^37^. Further variability is possibly related to data availability (Supplementary Fig. 6), which is mostly driven by differences in cloud coverage.

**Table 1:** Summary of NO_2_ changes in 2022 for selected cities and industrial areas in Ukraine. NO_2_ changes for the second quarter (April–June) of 2022 as compared to the same quarter in the reference period 2018–2021 based on S5P/TROPOMI observations as shown in Fig. 3. NO_2_ changes under calm wind condition (wind speed smaller than 3 m/s) are also shown (as reported in Supplementary Fig. 5). Population data for 2021 are given by the State Statistics Service of Ukraine^61^.

Name	Latitude (°N)	Longitude (°E)	Population/Capacity (MW)	NO_2_ change (10^14^ molec/cm^2^)		NO_2_ relative change (%)	
				All	Calm	All	Calm
Kyiv	50.450	30.523	2,952,301	− 20.43	− 36.5	− 42.4	− 51.5
Kharkiv	49.988	36.233	1,421,125	− 9.41	− 14.2	− 31.3	− 35.8
Odesa	46.483	30.713	1,010,537	− 4.78	− 7.0	− 20.3	− 27.1
Lviv	49.843	24.032	717,273	− 3.92	+ 0.9	− 15.1	+ 3.1
Zaporizhzhia	47.860	35.150	710,052	− 10.15	− 17.1	− 31.1	− 33.9
Kryvyi Rih	47.845	33.384	603,904	− 13.27	− 22.9	− 43.7	− 56.5
Mykolaiv	46.967	32.000	470,011	− 3.67	− 7.3	− 20.7	− 35.0
Mariupol	47.141	37.581	425,681	− 8.21	− 15.1	− 36.1	− 48.1
Enerhodar/Zaporizka TPP	47.499	34.657	52,237/2,850 MW	− 11.97	− 18.0	− 46.7	− 54.1
Horishni Plavni	49.038	33.665	49,854	− 6.46	− 8.5	− 27.6	− 28.2
Luhansk power plant	48.748	39.263	1,425 MW	− 6.85	− 12.2	− 34.8	− 46.0
Vuhlehirska power plant	48.464	38.202	3,600 MW	− 12.44	− 28.6	− 42.1	− 64.4

The analysis of NO_2_ observations available from the Ozone Monitoring Instrument (OMI) enabled the comparison to a longer reference period before the COVID-19 pandemic (2015–2019). Based on OMI observations, Supplementary Fig. 7 (solid lines) shows that the 2022 spring–summer mean NO_2_ concentrations over the most polluted areas in Ukraine were well below the 2015–2019 period (− 32% in Kyiv, − 23% near Donetsk and − 24% in the Dnipro/Zaporizhzhia area) and below the normal year-to-year variability (10–12%). Also, considering the short reference period 2019–2021 (dashed lines in Supplementary Fig. 7) for OMI observations, only changes the observed reduction in 2022 by 1 percentage point.

**Figure 4:**
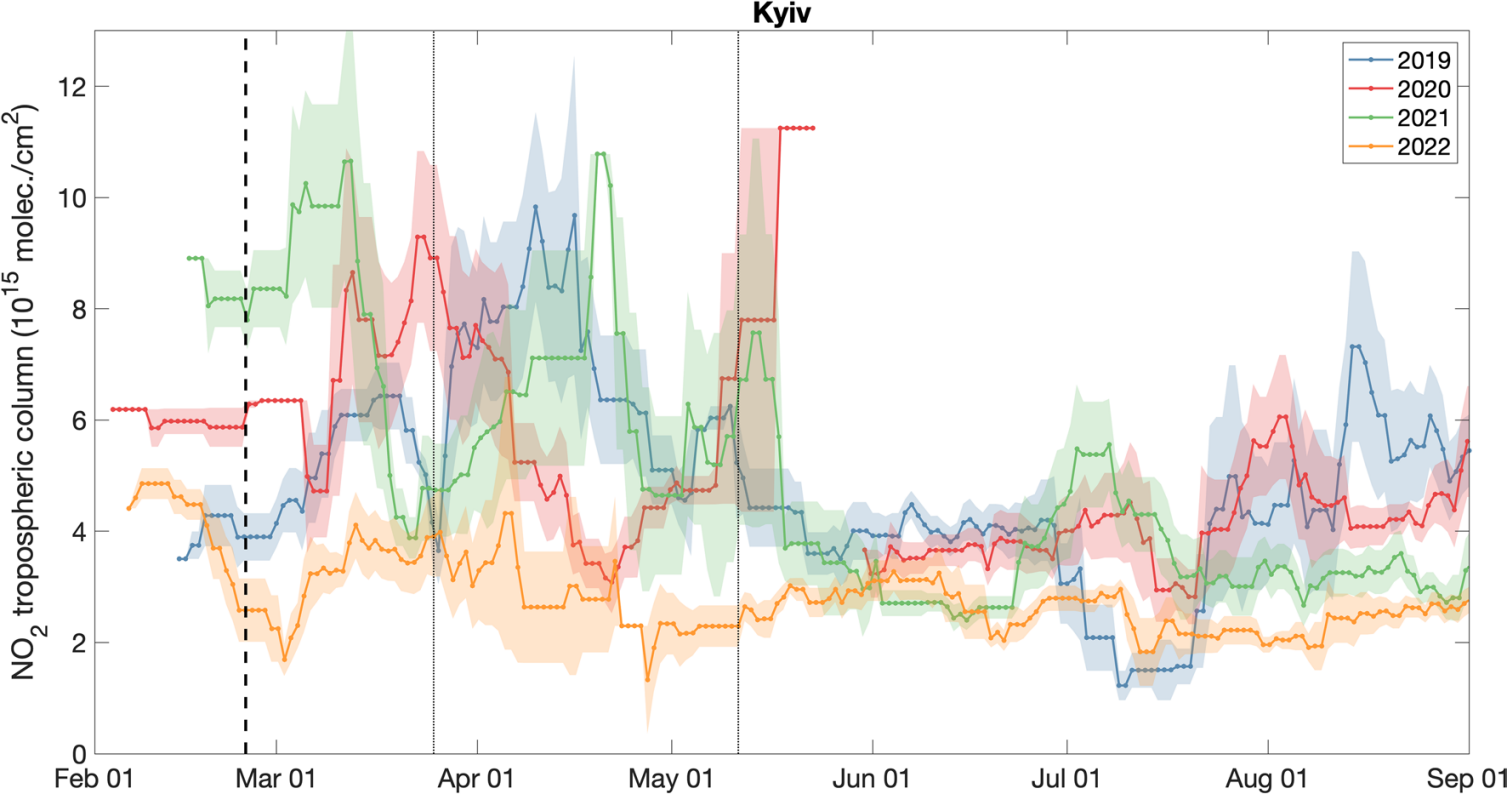
Timeseries of the 15-days running means of NO_2_ tropospheric columns during February-August. Colors correspond to different years (2019–2022). Shaded areas correspond to the standard error. Black dashed line indicates the beginning of the full-scale war on 24 February 2022, while the dotted lines correspond to the beginning and the end of the COVID-related restrictions in Kyiv in 2020. The NO_2_ reduction in 2022 (as compared to 2019 and 2021) persisted throughout the study period, while the NO_2_ concentrations in 2020 decreased for only about one month and rebounded to typical levels after the end of the COVID restrictions. The data gap in May 2020 is likely related to persistent cloudy conditions.

The observed NO_2_ reductions persisted for several months in 2022, unlike the short-term reduction observed during the COVID-19 restrictions in April 2020 (Fig. 4). The NO_2_ tropospheric column monthly averages over Kyiv in April 2022 were still about 40% smaller than those in 2020, and almost 60% smaller than 2019 and 2021. We note that changing meteorological conditions and data availability can affect the 15-days running mean NO_2_ values more than the quarterly NO_2_ anomalies. Also, the NO_2_ data availability is poorer during winter months due to lack of sunlight and frequent cloudy conditions, which yields higher variance in the running means (Fig. 4). The NO_2_ reductions reported here are generally larger than previously reported values^38^, possibly because we use consistent algorithm versions of the TROPOMI NO_2_ product throughout the study period.

Overall, we observed NO_2_ reduction over several cities, power plants and industrial areas in Ukraine. Some (Enerhodar, Mariupol, Vuhlehirska power plant) were occupied, others were close to the front line (Supplementary Fig. 1). The decrease in human activity during the war reduced electricity demand, which in turn reduced power plants’ production and thereby reduced NO_x_ emissions. In the bigger cities, the decrease in population has been likely driving the reduction in emissions from the transport and residential/commercial sector. A significant part of the population left the cities in the early days of the war, mostly from Kyiv and Kharkiv, and those who remained were subjected to curfew^39^. Also, freight transportation (as compared to the same period in 2021) decreased by 22% from road transport and 50% from railways^40^.

The World Bank reported a first estimate of significant economic decline in the country^4^. Ukraine’s Gross Domestic Product (GDP) declined in 2022 by over 30% year-on-year^41^. The State Statistics Service of Ukraine reported about 40–50% decline in industrial production in 2022^42^, especially from the steel and chemical industry that is concentrated in eastern Ukraine^43^. Such decline in industrial production led to a decrease in the use of electricity. According to the Ministry of Energy of Ukraine, Ukrainian industry reduced electricity consumption by 45% in 2022^44^. In particular, the metallurgical industry reduced electricity consumption by 52% (after the loss of the metallurgical giant in Mariupol), the chemical and petrochemical industry reduced consumption by 60%, the production of building materials by 47%, engineering by 38%, the food and processing industry by 24%, fuel industry by 24%, households by 16%. The total production of electricity in Ukraine in 2022 decreased by 27.5% compared to 2021 (production by thermal power plants decreased by 35%, and combined power-heat plants by 32%). The International Energy Agency also reported reductions in Ukraine’s power generation, including from coal-based energy sources^45^. These changes are consistent with the observed NO_2_ reductions, which can be linked to reductions in fossil fuel usage.

### Exceptional fire patterns near the front line

**Figure 5:**
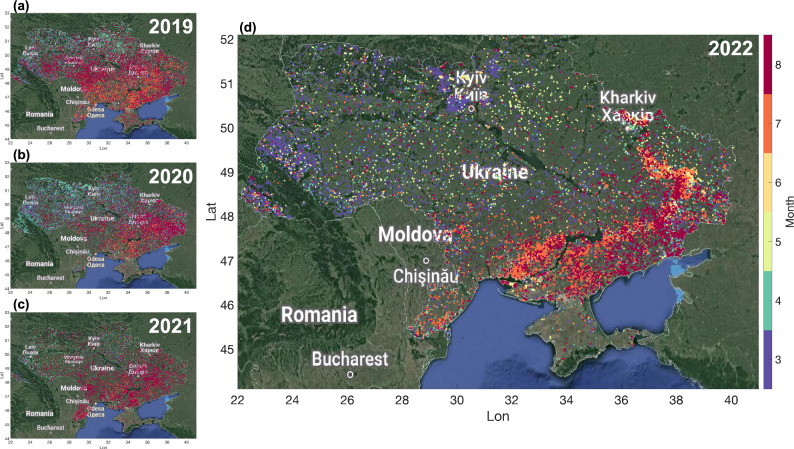
Fire detections from Suomi-NPP VIIRS over Ukraine between March and August for the years 2019–2022. Colors correspond to the month number. The maps were generated using the Matlab tool plot_google_map (Version 2.0.0.1, https://github.com/zoharby/plot_google_map) with administrative boundary shapefile from https://www.naturalearthdata.com/downloads/10m-cultural-vectors/10m-admin-0-countries/ (Version 3.1.0).

We also observe unique fire and thermal anomaly patterns in 2022. We analyzed the Suomi-NPP VIIRS data to further assess which areas are mostly disrupted by the war (Fig. 5). During the period 2019–2021 (before the full-scale war), the detected fires, which are generally attributed to crop harvesting or other vegetation fires, were distributed homogeneously over the Ukrainian territory (Fig. 5a-c) with most fires detected in March–April in north-western Ukraine and in July–August in the south-eastern part of the country. Some of the year-to-year variability is possibly related to changing cloud coverage and data availability. However, in 2022 most of the detected fires were located along the front line in the northern, eastern and southern parts of Ukraine, suggesting possible contribution from shelling (Fig. 5d). The 2022 spatio-temporal patterns are in line with the reported changes in the front line (Supplementary Fig. 1,^46^). For example, several fires were observed north-west of Kyiv (Supplementary Fig. 8) in the direction of Irpin-Ivankiv during March, when the Russian offensive focused on the area surrounding the Ukrainian capital (Supplementary Fig. 1). The Sentinel 2 false color images confirm the occurrence of several fires around Irpin on 21 March 2022 (Supplementary Fig. 9), just before the city was retaken by the Ukrainian troops ^47^. Towards the summer the fires are mostly detected along the front line in the eastern and southern parts of the country, which is in line with the location of the frontline at that time (Supplementary Fig. 1).

**Figure 6:**
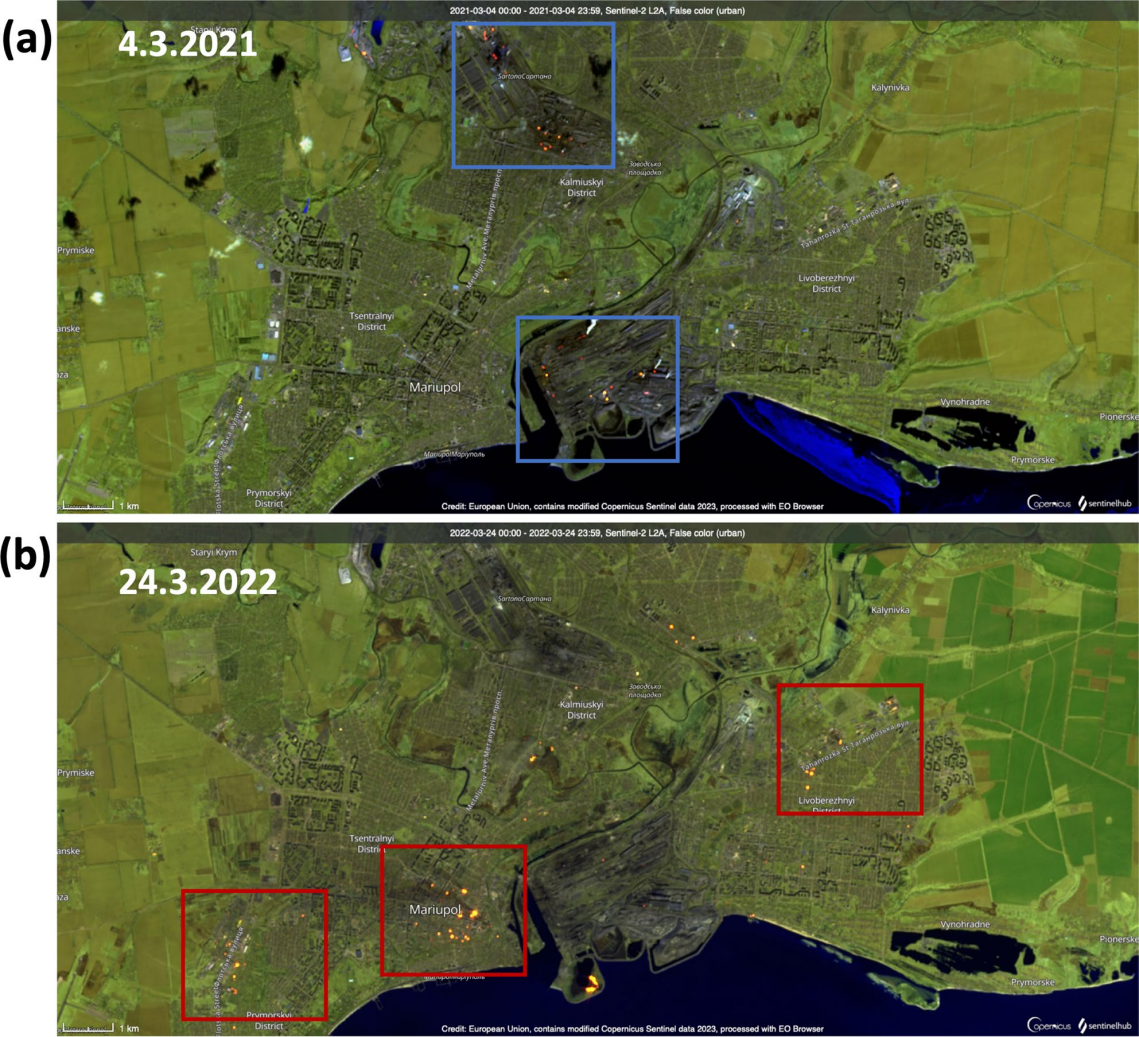
Sentinel-2 images over the city of Mariupol. S2 false color (urban) image on 4 March 2021 (**a**) and 24 March 2022 (**b**). Surfaces with elevated temperatures, such as fires and hot smoke, appear as red or yellow colors while vegetation is shown in green. Blue squares indicate the thermal anomalies related to metallurgic industry, while the red squares show the location of the fires possibly related to military activities. The satellite images were obtained from Sentinel Hub via EO Browser (https://apps.sentinel-hub.com/eo-browser/).

Satellite imagery data also provide snapshots of drastic changes in the fire or thermal anomaly patterns over the city of Mariupol, which was attacked during the first three months of the war (Fig. 6). In 2021, the Sentinel 2 false color images show thermal anomalies due to the hot smoke from the metallurgic industrial facilities (Fig. 6a), such as Azovstal and Mariupolsky Metal plant, and the Markohim coke plant. However, the thermal anomalies were no longer detected after the beginning of the full-scale war in March 2022 (Fig. 6b). This would suggest that an interruption of industrial activities occurred during the war. This is in line with the NO_2_ reduction observed in 2022 over the metallurgic industrial complex in the northern part of the city (Supplementary Fig. 10). The observed NO_2_ reduction can also be partially attributed to the reduced human activities due to the extensive evacuation of the city up until May 2022. On 24 March 2022, when the Russian offensive focused on Mariupol, larger and more intense clusters of fires were observed all around the city and countryside (Fig. 6b).

Similarly, a large cluster of fires was also observed in 2022 over the rural area around Velyka Oleksandrivka, near the front line and temporarily occupied territories north of Kherson (Supplementary Fig. 11b). This area was among those left unharvested due to the persistent shelling near the front line^8^. The unharvested wheat fields appear in the Sentinel 2 natural color images dark brown on 7 August 2022 (at the end of the harvesting season) as compared to the harvested (light brown) fields to the north and south (Supplementary Fig. 12b). In comparison, during the same day in 2020 the whole area appears homogeneous, without darker spots (Supplementary Fig. 12a), with fewer fires detected between March and August 2020 (Supplementary Fig. 11a), typically related to vegetation fires.

### CO_*2*_ anomalies indicate reductions in fossil fuel combustion

CO_2_ observations further explain the observed NO_2_ reductions over the areas most affected by the war. We find visual correspondence between the spatial patterns of the NO_2_ and CO_2_ anomaly differences, especially over areas with significant NO_2_ reductions. In particular, we observe a East–West gradient in the CO_2_ anomaly difference maps, with lower CO_2_ anomalies (as compared to 2021) over the eastern part of Ukraine (Fig. 2b), which is in line with the observed NO_2_ reductions. Since NO_x_ and CO_2_ are often co-emitted (e.g., from fossil fuel combustion), paired NO_2_ and CO_2_ anomaly reductions as those observed in eastern Ukraine indicate reductions in fossil fuel use in cities and industrial areas. On the other hand, slightly positive or less substantial NO_2_ and CO_2_ anomaly changes can be observed over western Ukraine. These differences are less localized than those observed in eastern Ukraine, where the largest emission sources are located, and can have large contribution from natural variability.

Because of the long lifetime, CO_2_ anomalies are more affected by atmospheric transport and not as localized as NO_2_. Due to the narrow swath of the OCO-2 instrument (width smaller than 10 km), the observed CO_2_ anomalies might not be able to fully capture year-to-year changes in CO_2_ emissions. Nevertheless, the expected reduction in fossil fuel usage is persistent in time over a relatively large region, which makes it possible to sufficiently cover the emission areas.

Additional NO_x_ and CO_2_ emissions compared to previous years are expected in the areas impacted by fires (Fig. 5d) as the result of military activity such as shelling, fires of petroleum products, buildings, and infrastructure objects, as well as forest fires and fires of agricultural fields. While the actual composition is unknown, military-related combustions could be fuel inefficient and might cause NO_x_ emissions in excess. Relatively small increases in NO_2_ can be observed in 2022 over some areas north-west of Kyiv, south-east of Kharkiv and in the south-eastern part of Ukraine (Fig. 2a) which were strongly affected by such fires, but the enhancements are difficult to attribute to a specific cause. Also, an increase in CO_2_ emissions is expected from the fires related to military activities near the front line. Military emissions though might be smaller than the emissions from different economic sectors in previous years, and appear to be partially hidden by the substantial reductions related to fossil fuel combustion in the average maps (Fig. 2) due to their temporary nature and sparse distribution, as well as the sparse coverage of the satellite observations.

## Discussion

Our results provide early insights into the magnitude of the impacts on human and industrial activities in Ukraine during the first stages of the full-scale war, but they remain in part qualitative. In addition to changes in emissions, concentrations of atmospheric gases depend on changing meteorological conditions, and further analysis will be needed to accurately estimate changes in air pollution and GHG emissions due to the war. Nevertheless, satellite-based observations make it possible to monitor such abrupt changes almost in real time, as compared to other methods that require longer time to collect all relevant information.

During peaceful times, NO_2_ reductions as those reported here would be considered as a welcome improvement of air quality and human health. In this case the observed changes tell a different story about the extent of the disruption caused by the war on the Ukrainian society. The NO_2_ reductions observed during March-August 2022 over power plants and other industrial areas reflect the reduction in industrial production and electricity demand from several industrial sectors, especially from the metallurgical and chemical industry. Drastic reductions in population (as well as changes in their mobility habits) have been reported for many Ukrainian cities, after many civilians moved to safer areas in the western part of the country or abroad^48^. The UN projects further population decline in 2023 and beyond^49^. The reductions in fossil fuel consumption in Ukraine might have been partly offset by an increase elsewhere^12^.

Overall, our analysis shows how the synergic use of Earth observations from different satellite platforms provides an important asset to monitor significant societal changes and has potential to support authorities in evaluating the impacts of the war on the Ukrainian society based on independent information. As presented in this study, the lack of monitoring capability in such situations as war and conflicts can be mitigated by combining satellite observations of trace gasses, which are now available with unprecedented resolution and accuracy, together with other satellite imagery and products. For example, satellite NO_2_ data can supplement and enhance NTL as indicator of human activity changes^50,51^, especially those not associated with lights or that happened during a power outage. While assessing the impacts of socio-economic changes using satellite observations generally requires expert analysis, new platforms and tools for data distribution and analysis are constantly developing and might enable faster and easier assessments in the future, also for non-expert users. Future instruments such as the European Copernicus CO2M (Anthropogenic Carbon Dioxide Monitoring^52^) and Japan’s GOSAT-GW^53^ missions will provide collocated observations of NO_2_ and CO_2_ which should enhance our ability of assessing changes in human activities during major socio-economic disruptions, as those presented here. Furthermore, atmospheric geostationary missions such as Sentinel-4^54^, Tropospheric Emissions: Monitoring of Pollution (TEMPO^55^), and Geostationary Environment Monitoring Spectrometer (GEMS^56^) will provide useful NO_2_ observations with multiple overpasses during one day.

Finally, the results shown here cover the time period between March-August 2022. Instead, beginning in the autumn of 2022, Russia launched regular missile attacks across the whole Ukraine to damage the energy infrastructure. This, in turn, affects industry and all spheres of life in Ukrainian society. Further studies should focus on the assessment of the full economic and environmental impacts throughout the war as well as during the reconstruction phase.

## Materials and methods

### TROPOMI and OMI NO_2_ product description and data processing

Nitrogen dioxide (NO_2_) is used as a proxy for human activities, as it is linked to fossil fuel combustion processes, and it is often co-emitted with carbon dioxide (CO_2_). Since NO_2_ is short-lived in the lower atmosphere, it can be found in high concentrations near the emission sources and can therefore be used to assess changes in emissions. Nitrogen oxides (NO_x_) are largely emitted as nitric oxides (NO), which rapidly transforms via photochemical reactions into NO_2_ (the species retrieved by satellite) until reaching a steady state.

TROPOMI is a passive-sensing hyperspectral nadir-viewing imager aboard the Copernicus Sentinel-5 Precursor (S5P) satellite, launched on 13 October 2017. S5P/TROPOMI NO_2_ retrievals are available at spatial resolution of 3.5 × 5.5 km^2^ (nadir) and daily global coverage, with overpass local time of 13:30. The NO_2_ retrievals are derived using TROPOMI’s UV–VIS spectrometer backscattered solar radiation measurements in the 405–465 nm wavelength range. Here, we use the reprocessed S5P-PAL NO_2_ dataset^57^, which includes a reprocessing of the TROPOMI NO_2_ data product from the start of the S5P mission until November 2021 using version 2.3.1 of the retrieval algorithm. Additionally, offline (OFFL) NO_2_ products are used to cover the period after November 2021.

The TROPOMI NO_2_ tropospheric columns were used to compile March-August average NO_2_ maps for the analysis of persistent NO_2_ enhancements and their year-to-year changes covering the period 2019–2022. TROPOMI NO_2_ retrievals are gridded into a regular 1 × 1 km^2^ grid using the area-weighted gridding method. Supplementary Fig. 6 shows the amount of data for each grid cell estimated from the sum of the weights used in the gridding. The weights (in the range 0–1) are defined for each orbit as the fraction of grid cell area covered by valid NO_2_ observations, with 1 corresponding to the grid cell fully covered. The amount of data is generally larger over water and lower near the mountains. The average maps for 2022 are based on about 21% (13%) more valid observations than for 2019 (2021) and about the same amount for 2020, mostly due to varying cloud conditions. In general, there are less valid NO_2_ retrievals during wintertime, due to frequent cloudy conditions and lack of sunlight.

We also derive the time series over the major cities and industrial sites in Ukraine, identified by the largest NO_2_ enhancements in the average maps in Fig. 1. We consider all the good quality TROPOMI NO_2_ observations within 4 km distance from the site of interest. We then derive quarterly averages anomalies as the difference between the quarterly averages for each year and the corresponding quarterly average for the reference period 2018–2021 (note that only third and fourth quarters are available for the year 2018). The quarterly anomalies are calculated to adjust for NO_2_ seasonal variability, as NO_2_ concentrations are generally higher in winter and smaller in summer, due to lower emissions and increased photo-chemistry in the summertime. The distance of 4 km from the source was chosen to avoid signals from neighboring sources to mix. The results do not substantially change when considering larger distance; for example, doubling the distance to the site to 8 km changes the quarterly differences reported in Table 1 by 0–5 percentage points. In order to reduce the effect of changing wind conditions, we calculate the NO_2_ anomalies considering only TROPOMI observations with corresponding wind speed smaller than 3 m/s (Supplementary Fig. 5). The 10-m wind components from the European Centre for Medium-Range Weather Forecasts—Integrated Forecasting System (ECMWF-IFS) are available within the TROPOMI NO_2_ product and are sampled according to the satellite pixels. Under calm wind conditions the NO_2_ enhancements are generally more pronounced and the spatial patterns are closer to the emission patterns, as pollution is not transported far away from the sources. Since satellite NO_2_ retrievals are only available under clear sky conditions, cloudiness will have a small effect except for changing the data availability (Supplementary Fig. 6).

In order to compare the NO_2_ levels in 2022 to a longer reference period, we use NO_2_ tropospheric columns from the Ozone Monitoring Instrument (OMI^58^) on board NASA’s Aura satellite, to derive spring–summer anomalies during the period 2015–2022 (as compared to the reference periods 2015–2019 and 2019–2021) over three areas of interest: Kyiv, Donetsk and the area around Zaporizhzhia. We use the OMI Level 3 gridded data available on a 0.25 × 0.25 degree grid. As compared to TROPOMI, OMI has similar measuring principle and overpass time, but lower spatial resolution (13 × 24 km^2^ in nadir). Therefore, OMI cannot capture the smallest spatial patterns near the emission sources but provides consistent NO_2_ observations since 2005. We limit the analysis of the spring–summer anomalies based on OMI data to the year 2015^20^ to avoid the potential effect of earlier NO_2_ reductions due to cleaner technologies or other emission changes^23,24^.

### OCO-2 data and XCO_2_ anomaly

Orbiting Carbon Observatory-2 (OCO-2^59^) is NASA’s CO_2_ observing mission launched in 2014. Each 0.333 s, OCO-2 provides 8 soundings with spatial footprint of 1.29 × 2.25 km^2^ across a swath less than 10 km wide. The column-averaged dry air mole fraction of CO_2_ (XCO_2_) is retrieved. Since CO_2_ is a long-lived greenhouse gas, it is transported and accumulated in the atmosphere. To extract the anthropogenic enhancements from such a large background signal, we calculate the XCO_2_ anomalies with respect to the daily medians for each 10-degree latitude band and linearly interpolated to each OCO-2 data point^60^. The CO_2_ anomalies are gridded into a regular 1 × 1 degree grid and averaged over the period March-August of each year. The averaged maps are generally based on 5–10 days of observations for each grid cell within this six-month period. The XCO_2_ anomaly difference maps are calculated with respect to the year 2021 (Fig. 2), as well as the period 2019–2021 (TROPOMI period) and 2017–2021 (OCO-2 period) (Supplementary Fig. 13). When changing the reference period, the spatial patterns in the difference map remain similar. Here we use the OCO-2 version V11r lite files, which include bias correction and data screening. The OCO-2 data product is available from NASA’s Goddard Earth Sciences Data and Information Services Center (GES DISC, https://disc.gsfc.nasa.gov).

### VIIRS active fire/thermal anomaly data

To identify the location of fires and thermal anomalies, we use the 375 m active fire data from the Visible Infrared Imaging Radiometer Suite (VIIRS), operating on-board the joint NASA/NOAA Suomi-National Polar-orbiting Partnership (S-NPP) satellite since October 2011, as available from NASA’s Fire Information for Resource Management System (FIRMS) archive. S-NPP VIIRS data are available twice a day at the equator with overpass times approximately 13:30 PM (ascending node) and 1:30 AM (descending node). The VIIRS fire detection algorithm is based on the 375 m middle and thermal infrared imagery data (I-bands). The product includes (among other variables) the time and coordinates of the fires as well as the corresponding fire radiative power (FRP, in MW). We used the active fire data product over Ukraine available in shapefile format from the FIRMS archive download: https://firms.modaps.eosdis.nasa.gov/download/.

### Sentinel 2 false color (urban) product

To provide context about the land properties and to further analyze the distribution of fires, we use enhanced natural color and false color images from Sentinel-2 as available from the Sentinel Hub EO Browser (https://apps.sentinel-hub.com/eo-browser). Sentinel-2 is a wide-swath multi-spectral imaging mission that provides high-resolution (10 m, 20 m, and 60 m, depending on the wavelength) images for 13 spectral bands in the visible and infrared wavelengths. The mission is based on a constellation of two identical satellites, Sentinel-2A and Sentinel-2B, both flying on a sun-synchronous orbit. The images have been available since March 2018 as 100 × 100 km^2^ tiles, with a maximum revisit time of 5 days (using both Sentinel-2A and 2B satellites). The Sentinel-2 mission is designed to monitor surface properties such as vegetation, soil and water cover. The false color (urban) products are based on the composite of bands 12, 11 and 4. Vegetation is visible in shades of green, while constructed areas are represented by white or gray. Snow and ice appear as dark blue, and water as black or blue. Surfaces with elevated temperatures, such as forest fires, calderas of volcanoes, gas flaring over oil and gas facilities as well as hot smoke from industrial activities, saturate the image in medium IR channels and appear in shades of red or yellow.

### Supplementary Information

Supplementary Figures.

## Data Availability

All satellite data used in this work are publicly available through NASA and ESA data distribution hubs. The S5P-PAL NO_2_ dataset reprocessed until November 2021 is available at: https://data-portal.s5p-pal.com. Additionally, the offline (OFFL) NO_2_ products used to cover the period after November 2021 is available from the Sentinel-5P Pre-Operations Data Hub (https://s5phub.copernicus.eu/). The OMI NO_2_ L3 data and the OCO-2 data can be accessed from NASA's GES DISC (https://disc.gsfc.nasa.gov). NASA VIIRS Active Fire data (SHP format) data set is provided by LANCE FIRMS and it is available online at: https://earthdata.nasa.gov/active-fire-data. Sentinel-2 images are available from the Sentinel Hub EO Browser (https://apps.sentinel-hub.com/eo-browser).
