# Supplementary material for: Satellites capture socioeconomic disruptions during the 2022 full-scale war in Ukraine
Source: Sci Rep. 2023 Sep 22;13:14954. doi: 10.1038/s41598-023-42118-w (PMC10516891; doi:10.1038/s41598-023-42118-w)
Supplement: Supplementary file 1 — Supplementary Figures. [file 41598_2023_42118_MOESM1_ESM.pdf]

Supplementary Materials for

Satellites capture socioeconomic disruptions during the 2022 full-scale war in Ukraine

Iolanda Ialongo,[1]* Rostyslav Bun,[2,3] Janne Hakkarainen[1], Henrik Virta[1], Tomohiro Oda[4,5,6]

[1] Space and Earth Observation Centre, Finnish Meteorological Institute, Helsinki, Finland
[2] Department of Applied Mathematics, Lviv Polytechnic National University, Lviv, Ukraine
[3] Department of Transport and Computer Science, WSB University, Dąbrowa Górnicza, Poland
[4] Earth from Space Institute, Universities Space Research Association, Washington, D.C., USA
[5] Department of Atmospheric and Oceanic Science, University of Maryland, College Park, MD, USA
[6] Graduate School of Engineering, Osaka University, Suita-city, Osaka, Japan

*Corresponding author. Email: iolanda.ialongo@fmi.fi

This PDF file includes:

Supplementary Figures 1 to 13

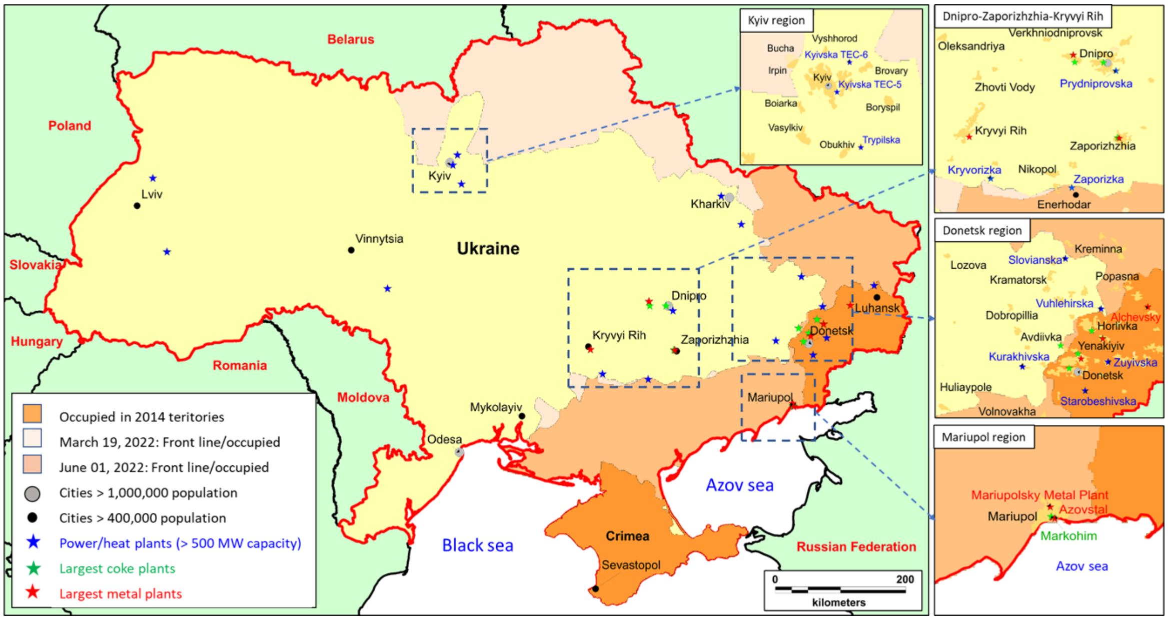

Supplementary Figure 1.

Map of Ukraine including the major cities and power plants as well as the occupied areas (Administrative maps: https://www.diva-gis.org).

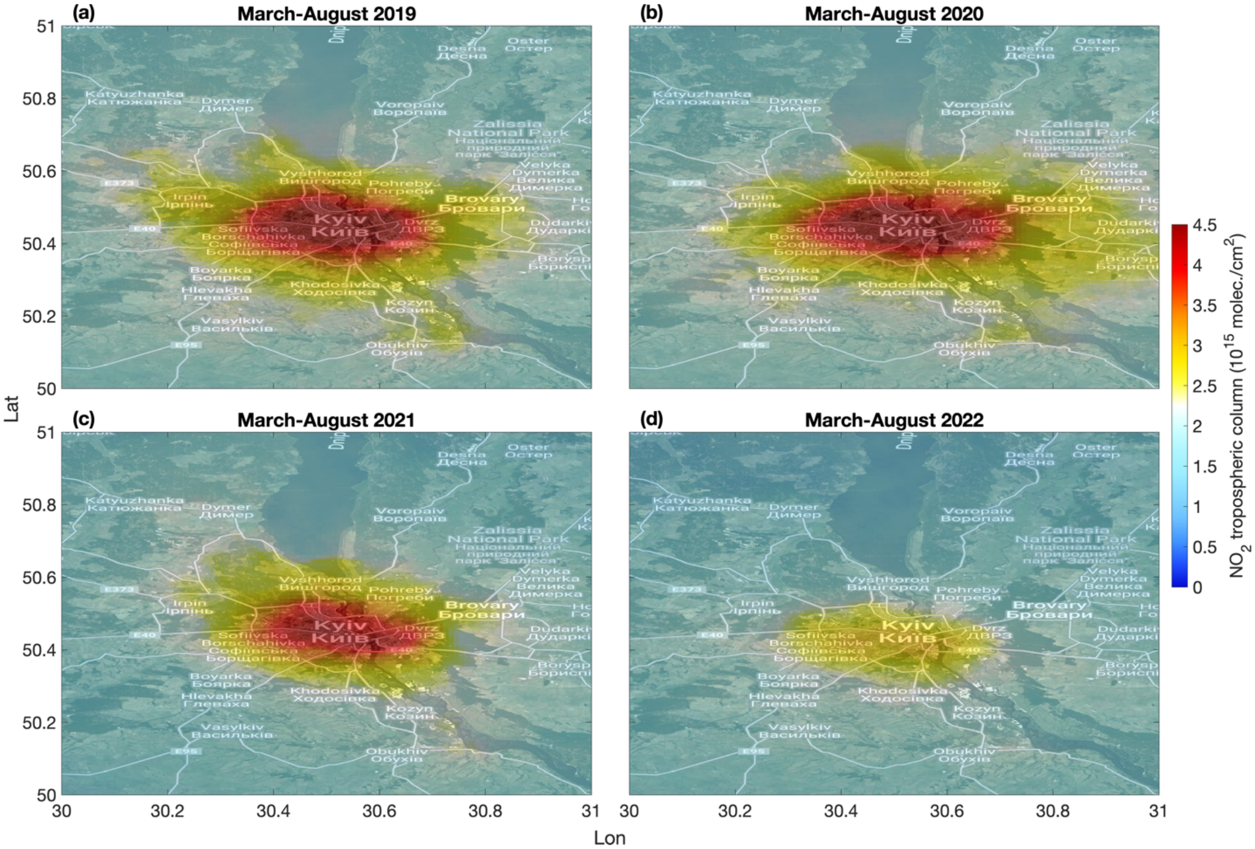

Supplementary Figure 2.

March-August average tropospheric NO_2 columns for each year between 2019 and 2022 over Kyiv based on S5P/TROPOMI data. The maps were generated using the Matlab tool plot_google_map (Version 2.0.0.1, https://github.com/zoharby/plot_google_map).

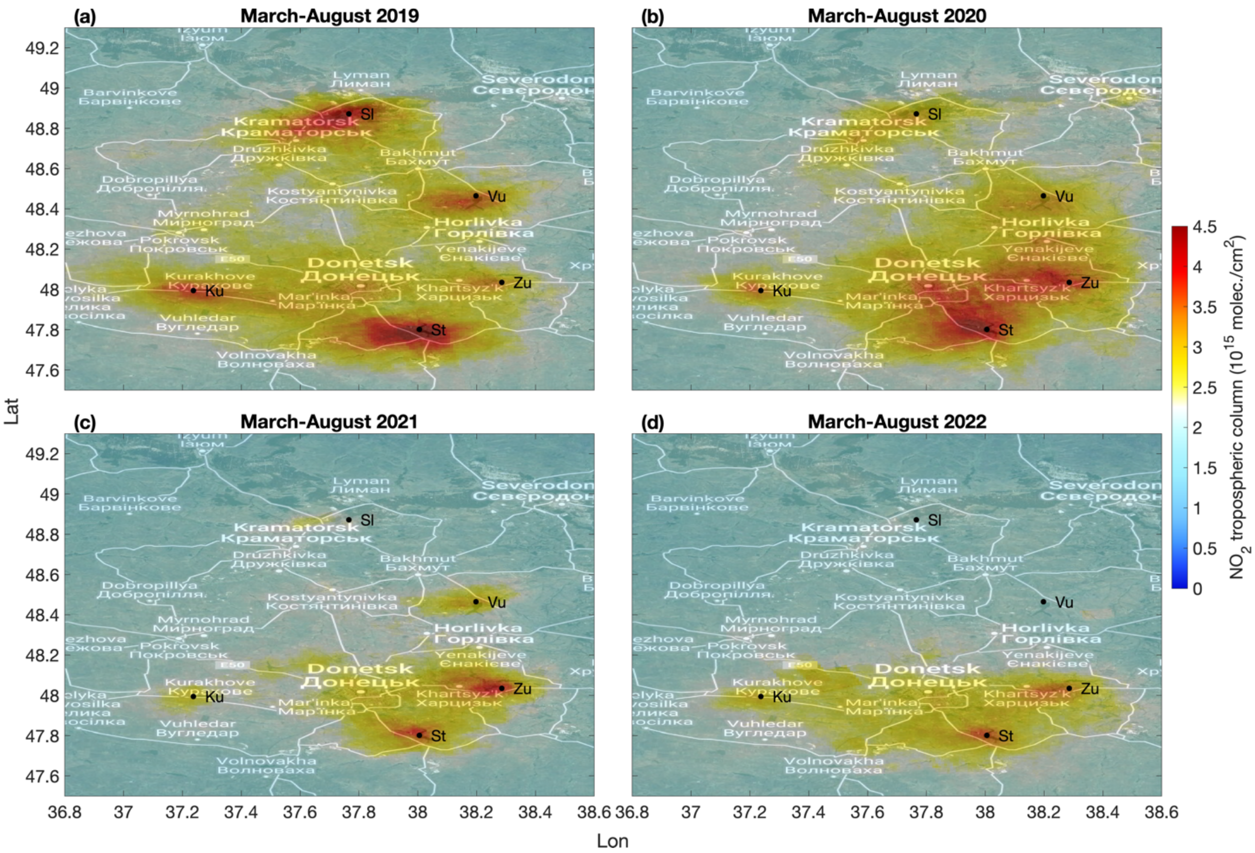

Supplementary Figure 3.

March-August average tropospheric NO_2 columns for each year between 2019 and 2022 over the Donetsk region based on S5P/TROPOMI data. St: Starobeshivska power plant, Sl: Slovyanska power plant, Ku: Kurakhivska power plant, Zu: Zuyivska power plant, Vu: Vuhlehirska power plant. The maps were generated using the Matlab tool plot_google_map (Version 2.0.0.1, https://github.com/zoharby/plot_google_map).

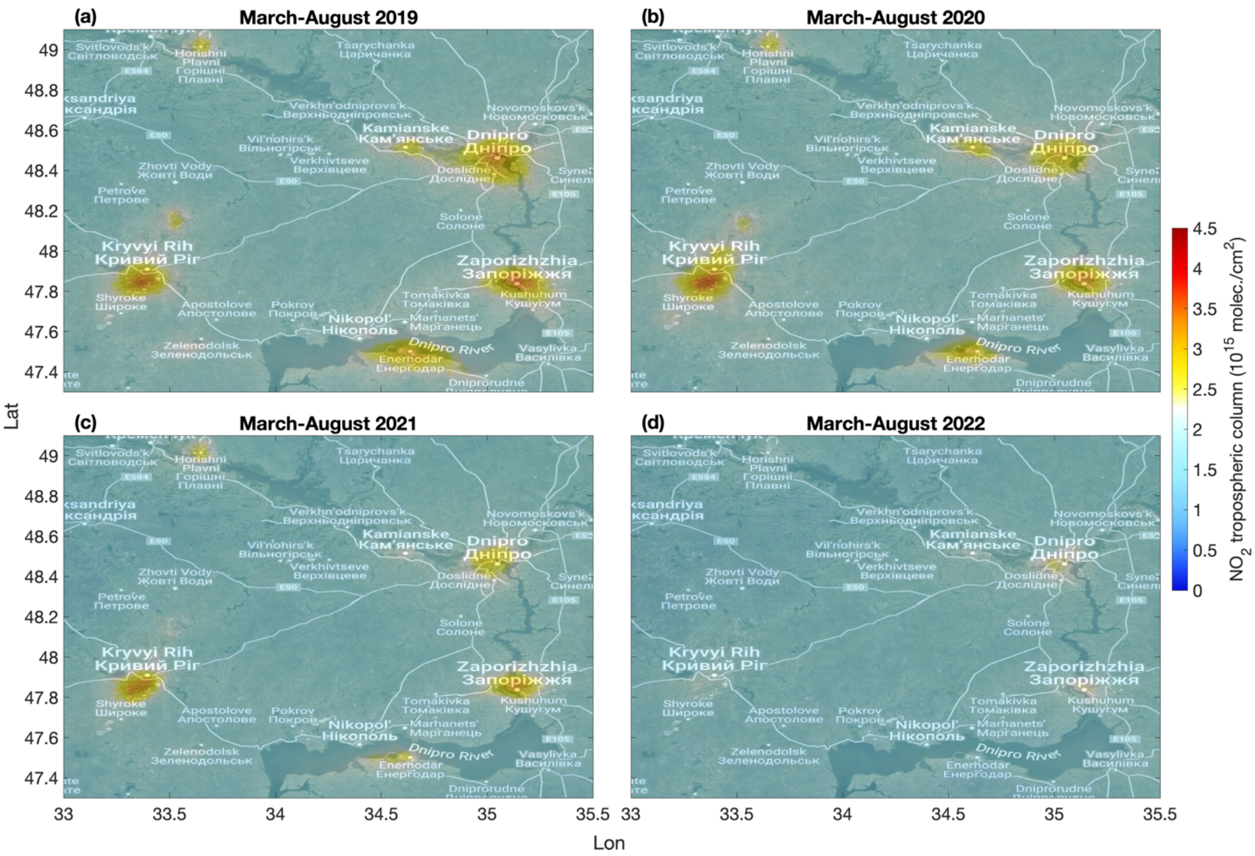

Supplementary Figure 4.

March-August average tropospheric NO_2 columns for each year between 2019 and 2022 over Dnipro, Zaporizhzhia, Kryvyi Rih based on S5P/TROPOMI data. The maps were generated using the Matlab tool plot_google_map (Version 2.0.0.1, https://github.com/zoharby/plot_google_map).

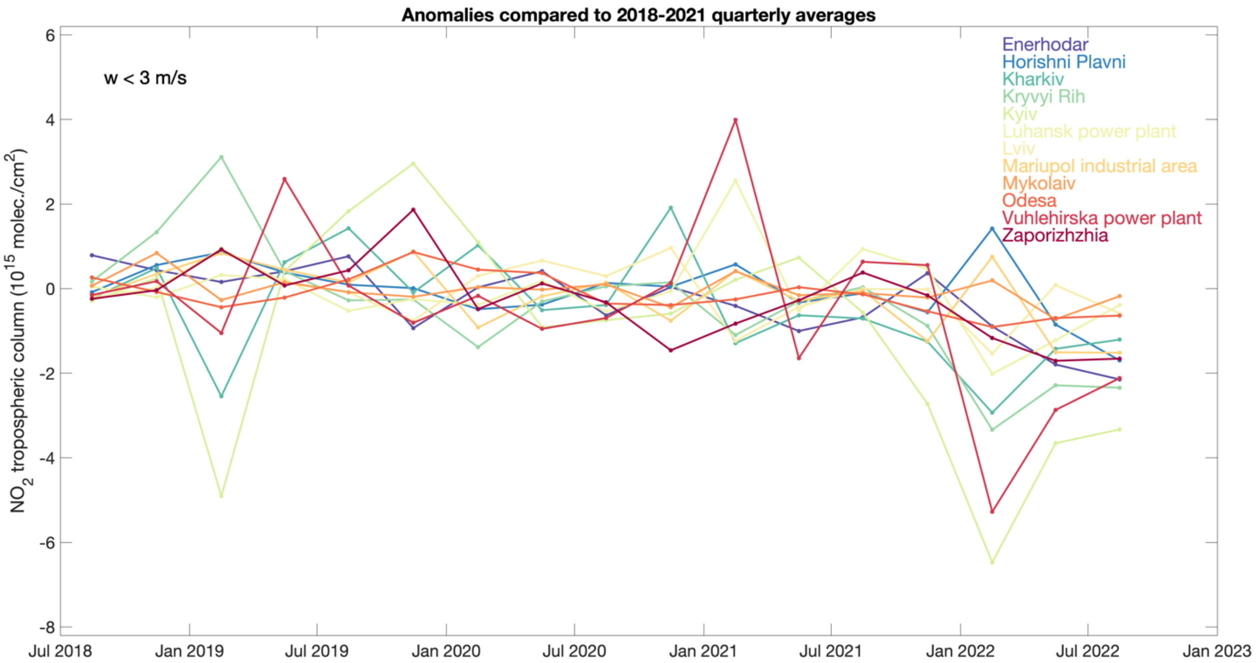

Supplementary Figure 5. Timeseries of the quarterly NO_2 anomalies under weak wind conditions (wind speed smaller than 3 m/s) for several cities and industrial facilities in Ukraine based on S5P/TROPOMI data. The anomalies are defined as the difference of the quarterly mean of individual years and the quarterly mean for the reference period 2018–2021.

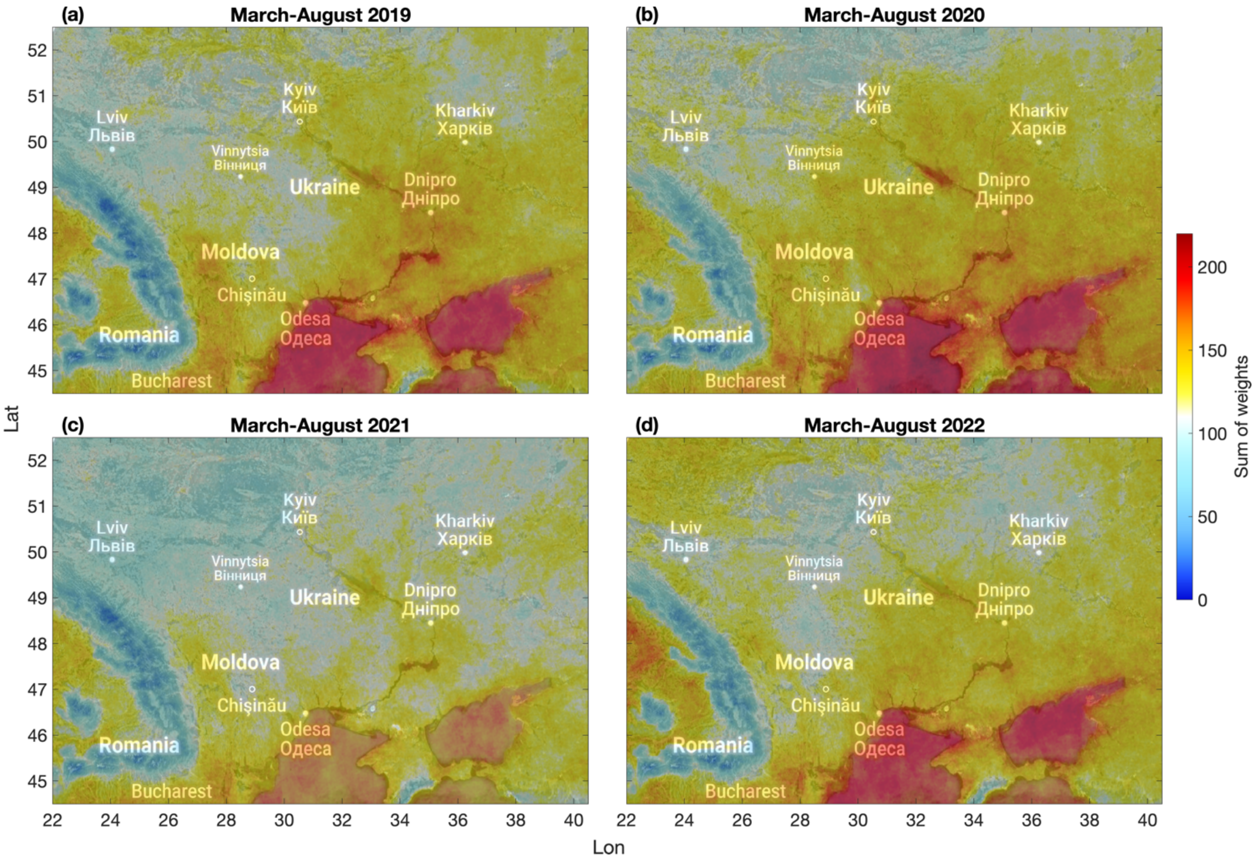

Supplementary Figure 6. Amount of TROPOMI NO_2 observations used in the spring-summer average maps in Fig. 1. The amount of data is estimated for each grid cell from the sum of the weights used in the gridding. The weights (in the range 0–1) are defined for each orbit as the fraction of grid cell area covered by valid NO_2 observations, with 1 corresponding to the grid cell fully covered. The maps were generated using the Matlab tool plot_google_map (Version 2.0.0.1, https://github.com/zoharby/plot_google_map).

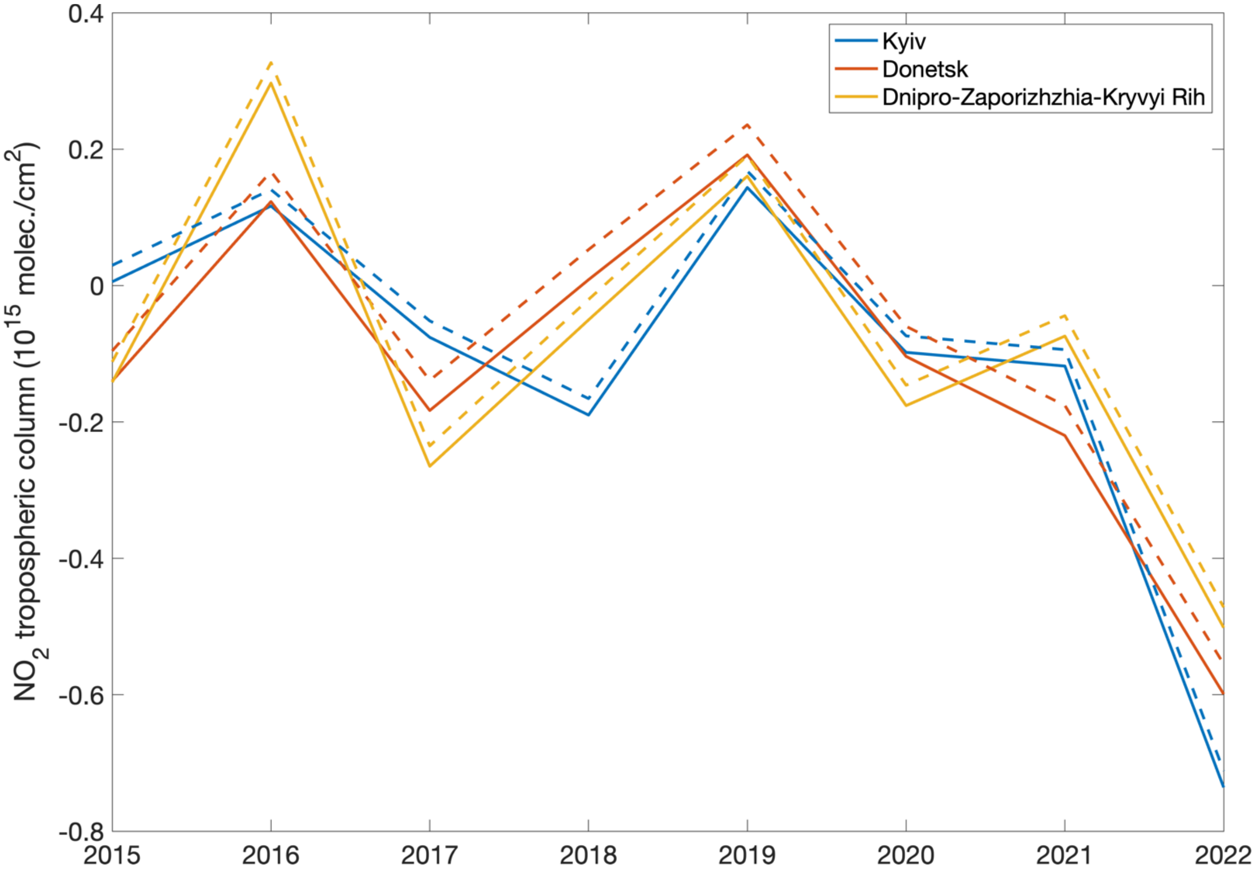

Supplementary Figure 7. Timeseries of the spring-summer (March-August) NO_2 anomalies for three polluted areas in Ukraine based on OMI observations. The anomalies are defined as the difference of the spring-summer of individual years and the spring-summer mean for the reference periods 2015–2019 (solid lines) and 2019–2021 (dashed lines).

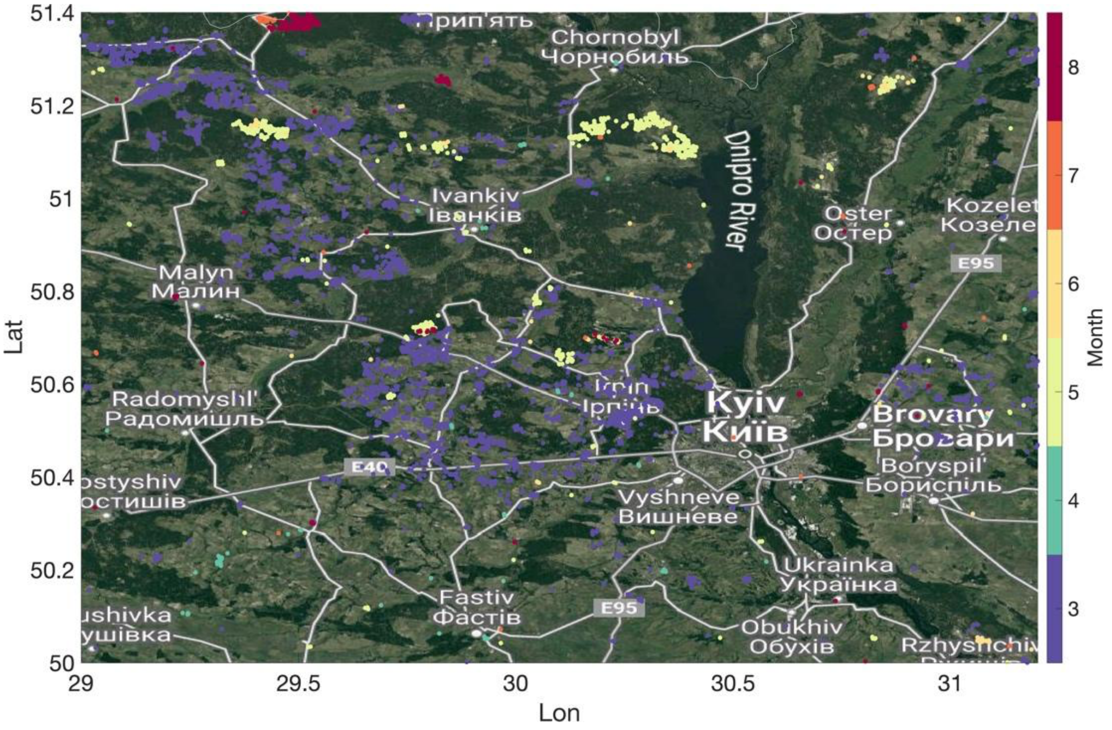

Supplementary Figure 8.

Fire detections from Suomi-NPP VIIRS over Kyiv between March and August for the year 2022. Colors correspond to the month number. The map was generated using the Matlab tool plot_google_map (Version 2.0.0.1, https://github.com/zoharby/plot_google_map).

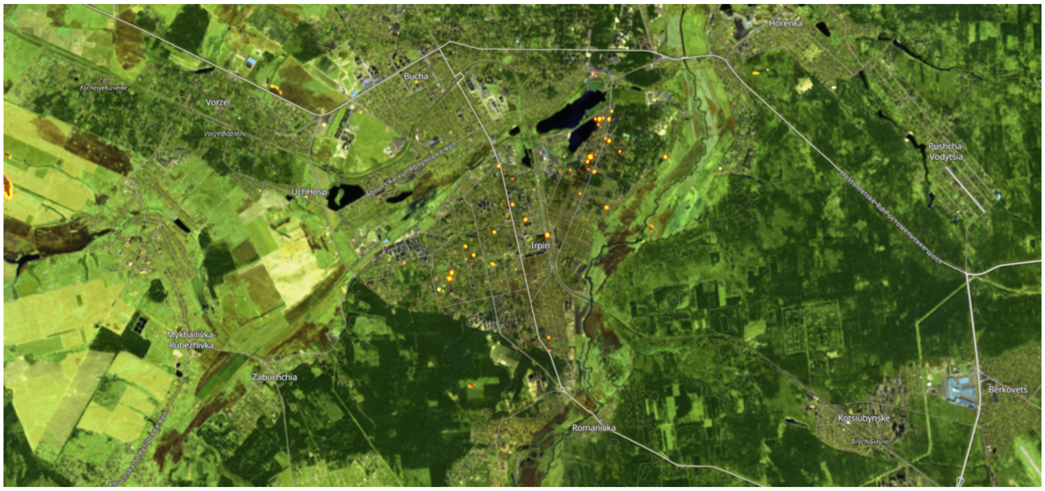

Supplementary Figure 9.

Sentinel-2 false color (urban) image over Irpin (NW of Kyiv) on 21 March 2022. The satellite image was obtained from Sentinel Hub via EO Browser (https://apps.sentinel-hub.com/eo-browser/).

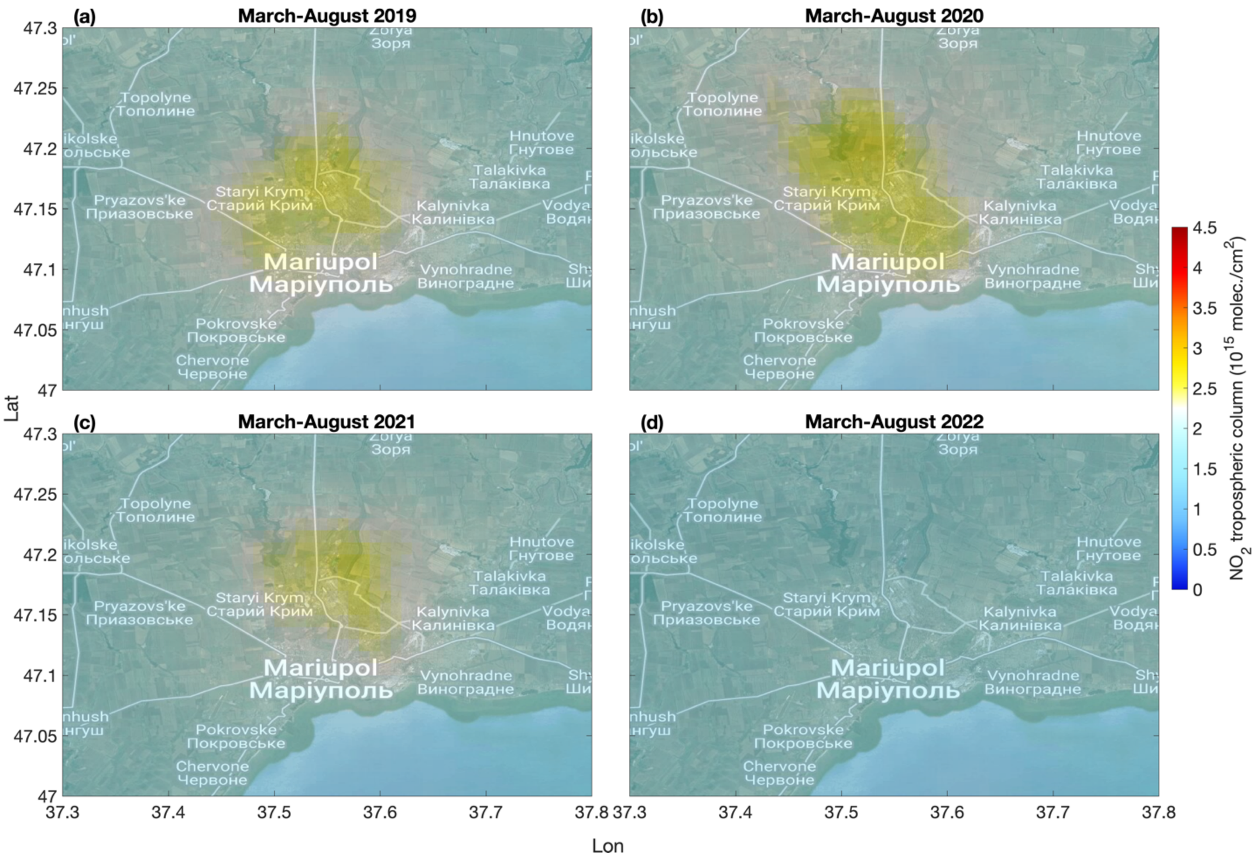

Supplementary Figure 10.

March-August average tropospheric NO_2 columns for each year between 2019 and 2022 over the city of Mariupol in south-eastern Ukraine based on S5P/TROPOMI data. The maps were generated using the Matlab tool plot_google_map (Version 2.0.0.1, https://github.com/zoharby/plot_google_map).

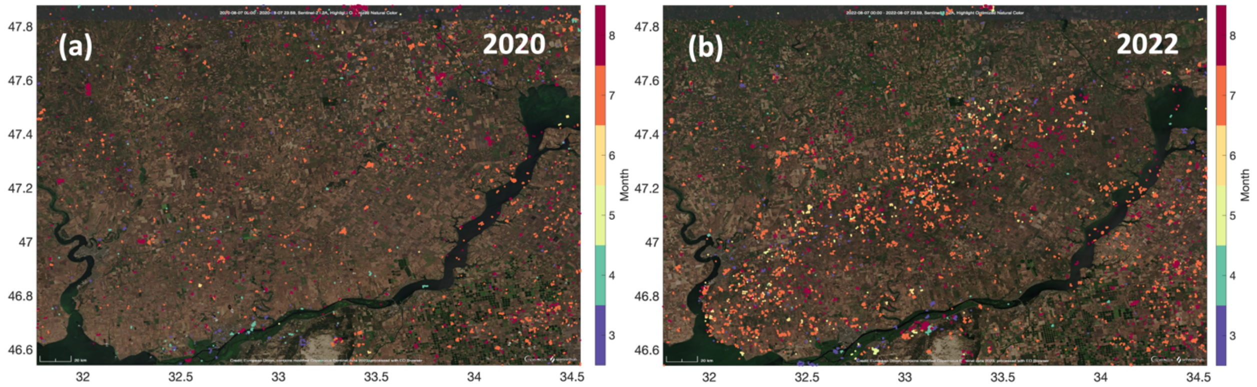

Supplementary Figure 11.

Sentinel-2 highlight-optimized natural color images on 7 August 2020 (a) and 2022 (b) overlayed with all fires/thermal anomalies from VIIRS on Suomi-NPP detected during March-August of each year. The satellite images were obtained from Sentinel Hub via EO Browser (https://apps.sentinel-hub.com/eo-browser/).

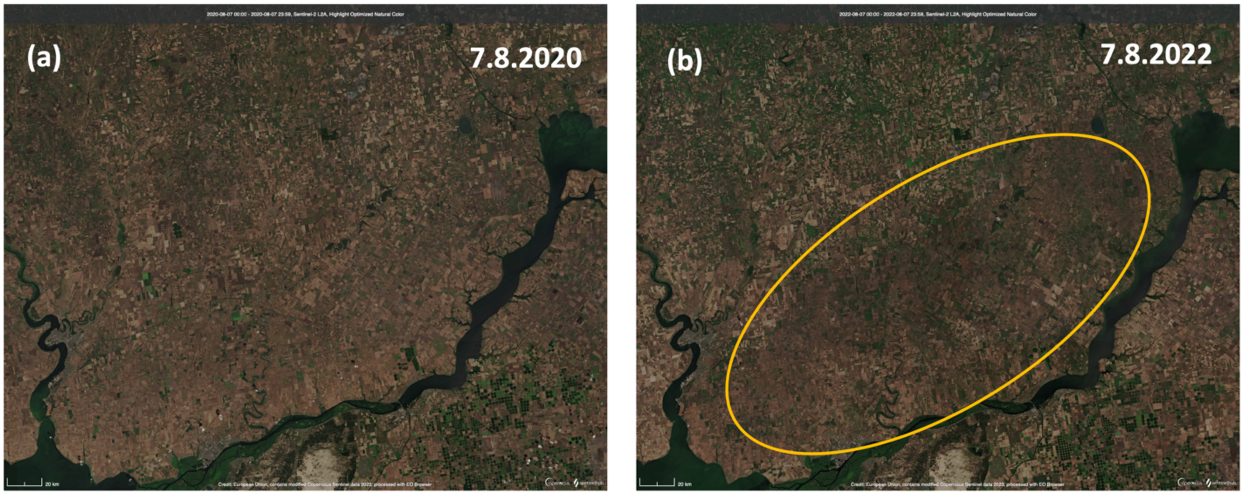

Supplementary Figure 12.

Sentinel-2 highlight-optimized natural color images on 7 August 2020 (a) and 2022 (b). The yellow circle indicates the darker area near the front line that was not harvested in 2022. The satellite images were obtained from Sentinel Hub via EO Browser (https://apps.sentinel-hub.com/eo-browser/).

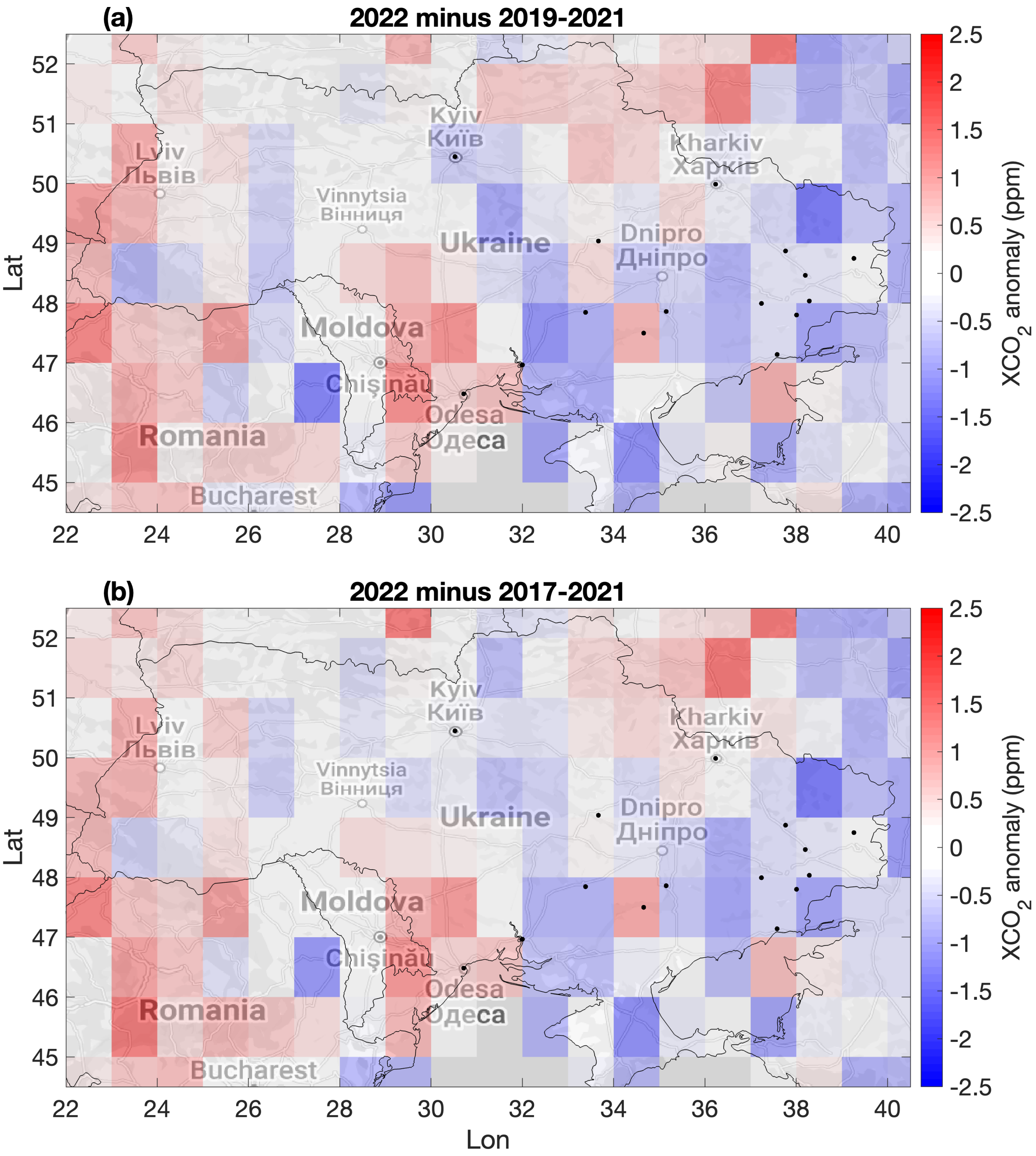

Supplementary Figure 13. XCO_2 anomaly difference maps over Ukraine. (a) Difference of the March-August mean XCO_2 anomalies (defined as the difference from the latitudinal background) between 2022 and the 2019-2021average based on OCO-2 observations. (b) Difference of the March-August mean XCO_2 anomalies between 2022 and 2017–2021 based on OCO-2 observations. Blue colors indicate reductions observed in 2022. Black dots correspond to the major cities, industrial areas and power plants. The maps were generated using the Matlab tool plot_google_map (Version 2.0.0.1, https://github.com/zoharby/plot_google_map) with administrative boundary shapefile from https://www.naturalearthdata.com/downloads/10m-cultural-vectors/10m-admin-0-countries/ (Version 3.1.0).
